# Protective effects of alpha lipoic acid on radiation-induced salivary gland injury in rats

**DOI:** 10.18632/oncotarget.8661

**Published:** 2016-04-09

**Authors:** Jin Hyun Kim, Kyung Mi Kim, Myeong Hee Jung, Jung Hwa Jung, Ki Mun Kang, Bae Kwon Jeong, Jin Pyeong Kim, Jung Je Park, Seung Hoon Woo

**Affiliations:** ^1^ Biomedical Research Institute, Gyeongsang National University Hospital, Jinju, Gyeongnam, Republic of Korea; ^2^ Institute of Health Science, Jinju, Gyeongnam, Republic of Korea; ^3^ Department of Otolaryngology, Jinju, Gyeongnam, Republic of Korea; ^4^ Department of Internal Medicine, Jinju, Gyeongnam, Republic of Korea; ^5^ Department of Radiation Oncology, Gyeongsang National University School of Medicine and Gyeongsang National University Hospital, Jinju, Gyeongnam, Republic of Korea

**Keywords:** alpha lipoic acid, salivary gland, radiation, Nox-2, complication

## Abstract

**Purpose:**

Radiation therapy is a treatment for patients with head and neck (HN) cancer. However, radiation exposure to the HN often induces salivary gland (SG) dysfunction. We investigated the effect of α-lipoic acid (ALA) on radiation-induced SG injury in rats.

**Results:**

ALA preserved acinoductal integrity and acinar cell secretary function following irradiation. These results are related to the mechanisms by which ALA inhibits oxidative stress by inhibiting gp91 mRNA and 8-OHdG expression and apoptosis of acinar cells and ductal cells by inactivating MAPKs in the early period and expression of inflammation-related factors including NF-κB, IκB-α, and TGF-β1 and fibrosis in late irradiated SG. ALA effects began in the acute phase and persisted for at least 56 days after irradiation.

**Materials and Methods:**

Rats were assigned to followings: control, ALA only (100 mg/kg, i.p.), irradiated, and ALA administered 24 h and 30 min prior to irradiation. The neck area including the SG was evenly irradiated with 2 Gy per minute (total dose, 18 Gy) using a photon 6-MV linear accelerator. Rats were killed at 4, 7, 28, and 56 days after radiation.

**Conclusions:**

Our results show that ALA could be used to ameliorate radiation-induced SG injury in patients with HN cancer.

## INTRODUCTION

Radiation therapy is used conventionally to manage head and neck tumors. Exposure of the salivary gland to radiation during head and neck radiotherapy is often unavoidable. Xerostomia induced by salivary gland damage is a representative complication of irradiation treatment for head and neck cancers and is typically irreversible and a general health problem in patients with head and neck cancers [1–4]. It is clinically important to prevent radiation-induced death of off-target cells when treating patients with cancer. Moreover, irradiation-induced salivary gland hypofunction not only affects patient quality of life but also interrupts radiotherapy and tumor control. Thus, salivary damage and consequent xerostomia are serious questions in the oral/or head and neck- and radio-oncology fields.

Salivary flow can decrease markedly within the first weeks during radiotherapy and up to months and even years after radiotherapy [5–7]. The human parotid gland is more radiosensitive compared to the submandibular gland. [8–10] More than a 50% reduction in parotid gland function has been reported within a few days after exposure of the head and neck region to low doses of irradiation [11]. In contrast, the parotid and submandibular glands respond similarly with reduced flow rate, increase in the lag phase and a reduced gland weight and number of acinar cells after single dose irradiation [5]. Above all, much less attention has been given to the submandibular gland than the parotids, and the role of the submandibular salivary gland in radiation-induced xerostomia has been minimized. Single dose irradiation was examined in this study; therefore, our data focused on the submandibular gland.

An increasing number of trials have been conducted on restoring radiation-induced salivary gland hypofunction using various treatments, such as cell therapy [12–14] cytokines [15], or antioxidants [16]. However, the effect of α-lipoic acid (ALA) on minimizing salivary gland damage has not been documented. ALA is a strong antioxidant with high reactivity to free radicals that facilitates regeneration of vitamins C and E and elevates tissue levels of glutathione [17]. ALA protects mouse hematopoietic tissues against radiation injury and increases the LD50 from 8.6 to 10.9 Gy with a dose-reduction factor of 1.26 [18]. Furthermore, a 28-day ALA treatment lowered lipid peroxidation in children chronically exposed to low doses of radiation by the Chernobyl nuclear accident [19].

The purpose of this study was to investigate the radioprotective potential of ALA on the salivary gland and the possible mechanisms by which ALA exerts radioprotection in a rat model.

## RESULTS

### Effects of ALA on radiation-induced pathophysiological changes

**Figure 1:**
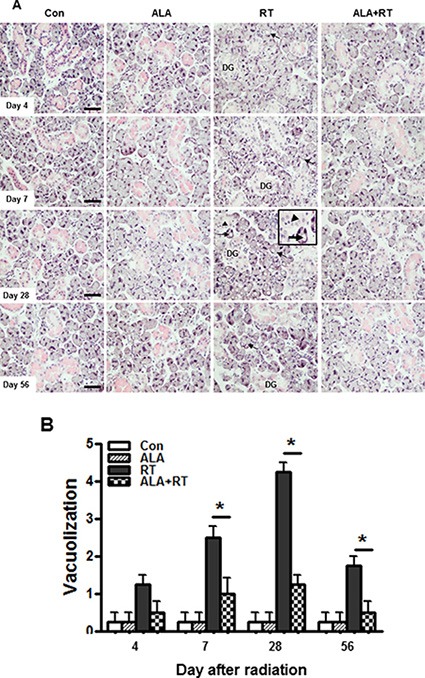
ALA decreases pathological changes in the salivary gland after irradiation Histopathological changes of the submandibular gland 4, 7, 28, and 56 days after irradiation. All salivary gland tissue were sectioned and stained with H & E. Exposing the salivary gland to irradiation resulted in the vacuolization of acinar cells (arrows) and the vacuolization decreased in the ALA treatment group before irradiation (**A**). Injury scoring was based on the number of injured acinar cells with cytoplasmic vacuoles in x 200 magnification field. Injury grading is described in the “Materials and Methods” section (**B**). Con, control. ALA, ALA only. RT, irradiated. ALA + RT, received ALA before irradiation. Scale bar, 50 μm. Each bar shows the mean ± SEM; *p < 0.05 indicates differences between RT and ALA + RT groups. Con (*n* = 3), ALA (*n* = 3), RT (*n* = 10), ALA + RT (*n* = 10).

Normal acinar and ductal cells was observed in the control or ALA only groups and any of the time points. A considerable loss of granulation of granular convoluted tubule cells (DG) and acinar cells with cytoplasmic vacuoles were started to observe 4 day after irradiation (arrow. Figure 1A). Pyknotic nuclei was shown in the irradiated SG on 28 day (arrowhead. Figure 1A). Much clear pyknotic nuclei was found in the serous acinar (arrow. Supplementary Figure 1A), the mucous acinar (arrow. Supplementary Figure 1B), and the ductal cells (arrow. Supplementary Figure 1C) in the irradiated SG. The ALA pre-treatment significantly prevented the radiation-induced acinar vacuolization on 7, 28, and 56 day after irradiation (Figure 1B).

### Effects of ALA on salivary gland dysfunction

**Figure 2:**
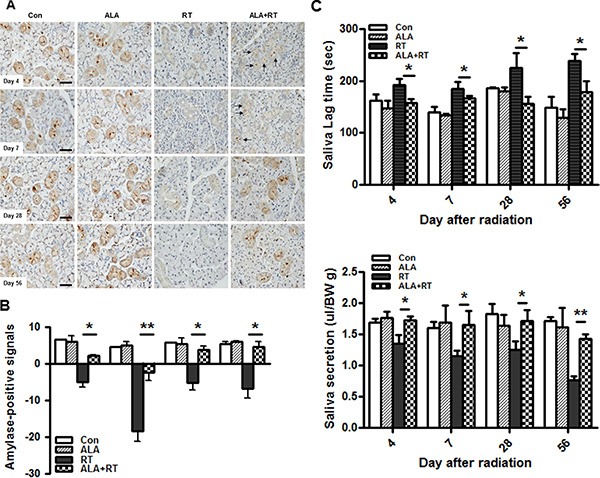
ALA ameliorates radiation-induced salivary dysfunction The function of acini was evaluated by amylase staining in the submandibular gland at 4, 7, 28, and 56 days after radiation (**A**). Amylase content decreased significantly in the irradiated gland and ALA-treated SG showed much denser signals than that of the irradiated SG at all days (**A** and **B**). Saliva lag time was examined as the first time to secret the saliva from pilocarpine stimulation and secretion is expressed as the total output of saliva collected normalized to body weight after pilocarpine stimulation. The ALA-treated group showed improved lag time and increased saliva secretion relative to the irradiated group (**C**). Con, control. ALA, ALA only. RT, irradiated. ALA + RT, received ALA before irradiation. Scale bar, 50 μm. Each bar shows the mean ± SEM; *p < 0.05 indicates differences between RT and ALA + RT groups. Con (*n* = 5), ALA (*n* = 5), RT (*n* = 15), ALA + RT (*n* = 15).

To determine whether administering ALA improved salivary function, immunochemical staining for amylase was carried out and saliva lag time and saliva secretion were measured at each day after irradiation. The irradiated group showed significantly reduced amylase content compared with that of the control and ALA alone group on all days after radiation. A slight increase in the amylase-positive signals was detected in ALA-treated SG compared to irradiated group on day 4 and 7 but the difference was significant. Remarkably, more amylase content-containing acini appeared to be more numerous in ALA-treated SG than in irradiated SG on day 28 and 56 (Figure 2A and 2B). Saliva lag time and saliva secretion were measured at all time points. The lag time of salivation was significantly prolonged in the irradiated group and was significantly improved in ALA-treated SG (Figure 2C). Saliva production was also improved in ALA-treated SG compared to the irradiated group (Figure 2C).

### Effects of ALA on oxidative stress

**Figure 3:**
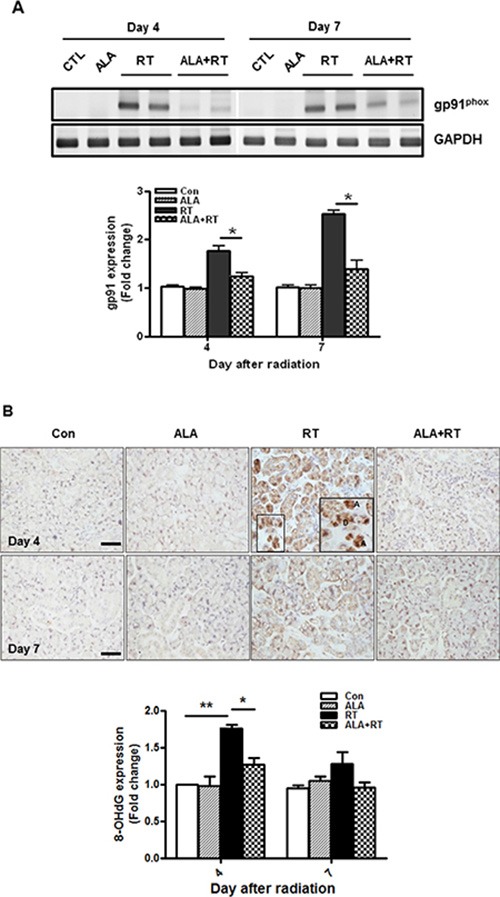
ALA reduces radiation-induced oxidative stress via Nox2 and 8-OHdG gp91 mRNA expression was determined by RT-PCR. gp91 mRNA expression decreased in the ALA-treated irradiated group compared to that in the irradiated group on 4 and 7 days after radiation (**A**). 8-OHdG-positive signals increased significantly in the salivary gland after irradiation and less intense signals were detected in the ALA-treated irradiated group (ALA + RT) than those in the irradiated group 4 days after radiation (**B**). Con, control. ALA, ALA only. RT, irradiated. ALA + RT, received ALA before irradiation. Scale bar, 50 μm. Each bar shows the mean ± SEM; *p < 0.05 and **p < 0.01 indicates differences between RT and ALA + RT groups. The fold changes are calculated as the ratio of the final value in the each group to the value in control group at day 4 (set as “1”). Con (*n* = 3), ALA (*n* = 3), RT (*n* = 10), ALA + RT (*n* = 10).

Radiation is known to enhance production of reactive oxygen species (ROS) that induce oxidative damage in the submandibular glands [20]. Nicotinamide adenine dinucleotide phosphate oxidase (Nox) expression is critical for ROS production and six Nox family were identified [21]. Of the family, no studies have shown Nox2, also termed gp91^phox^, involvement in radiation-induced SG injury compared to other members. We determined whether ALA is related to gp91 mRNA expression. RT-PCR data demonstrated that gp91^phox^ mRNA was well expressed in the irradiated SG on day 4 and 7 after radiation. The expression was significantly decreased by ALA treatment on day 4 and 7 (Figure 3A). But no gp91^phox^ expression was detected by the irradiation on days 28 and 56 (data not shown). Immunohistochemical staining of 8-OHdG, a ROS-induced DNA damage marker, was performed to investigate effect of ALA on radiation-induced oxidative stress. 8-OHdG-positive signals were detected in the nuclei of irradiated acinar cells (“A” in Figure 3B) and ductal cells (“D” in Figure 3B) and were much denser on day 4 than on other days. The positive signals for 8-OHdG were confirmed in the Supplementary Figure 2. This signal was decreased on day 4 by ALA treatment and this signal tended to reduce in ALA-treated SG compared to control group, but was not significant on day 7 (Figure 3B). 8-OHdG-positive cells were slightly found in irradiated SG on days 28 and 56 (data not shown), but were not as prominent compared to day 4 and 7.

### Effects of ALA on apoptosis

**Figure 4:**
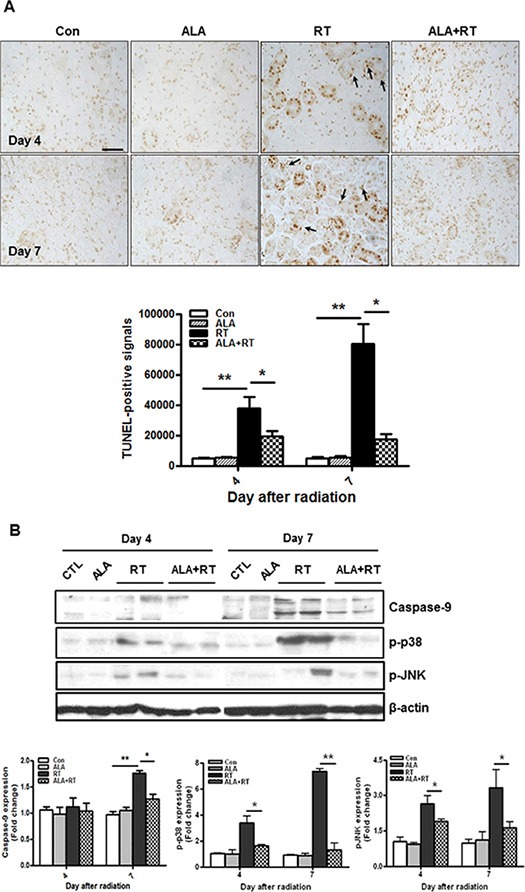
ALA decreases radiation-induced cell death in the SG Cell death was analyzed with the TUNEL assay. Microscopic image shows increased cell death in the RT group compared to the control and ALA-treated irradiated groups (ALA+RT) 4 and 7 days after irradiation. Scale bar, 50 μm (**A**). Western blotting was performed using anti-p-p38, anti-p-JNK. Expression levels of p-p38 and p-JNK were induced in the RT group compared to that in the control and ALA-treated irradiated groups (ALA + RT) 4 and 7 days after irradiation. β-actin was used as the loading control (**B**). Con, control. ALA, ALA only. RT, irradiated. ALA + RT, received ALA before irradiation. Representative blots are derived from three separate experiments. Values are represented as the mean ± SEM. *P < 0.05 and **p < 0.01 indicates differences between RT and ALA + RT groups. The fold changes are calculated as the ratio of the final value in the each group to the value in control group at day 4 (set as “1”). Con (*n* = 3), ALA (*n* = 3), RT (*n* = 10), ALA + RT (*n* = 10).

Terminal deoxynucleotidyl transferase dUTP nick end labeling (TUNEL) staining was performed to investigate the mechanisms for apoptosis. TUNEL-positive cells are found both the ductal cells and the acinar cells (arrow. Figure 4A). The highest level of apoptosis in the irradiated SG was observed on 7 days (Figure 4A). ALA significantly suppressed early induction of apoptosis (4 and 7 days) compared to irradiated rats at the same time points. The level of apoptosis returned to untreated levels on days 28 and 56 but no significant differences were observed between irradiated rats and ALA-treated rats 28 and 56 days after radiation (data not shown). However, TUNEL may be not reliable in salivary glands due to endogenous enzymatic activity. So, to confirm anti-apoptotic effect of ALA in the irradiated SG, caspase-9 cleavage and mitogen activated protein kinase (MAPK) levels, are important signaling cascades that mediate cellular functions including apoptosis [23, 24], were investigated. Figure 4B showed that radiation significantly increased caspase-9 cleavage and activation of p-38 and JNK on day 4 and 7 after radiation. However, these expression levels were significantly decreased 4 and 7 days in the ALA group comparison to that in the irradiated group. Interestingly, similar to gp91 mRNA expression, caspase-9 cleavage and activation of p-38 and JNK were not found on days 28 and 56 in all groups (data not shown).

### Effect of ALA on inflammation and fibrosis

**Figure 5:**
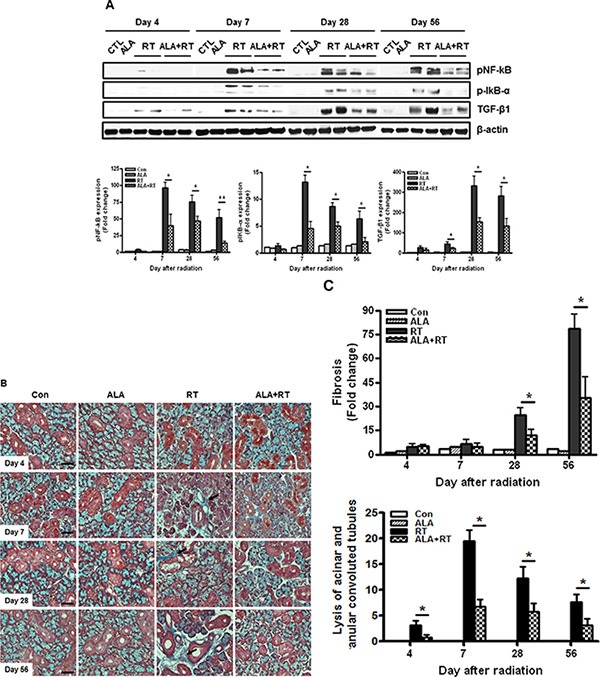
ALA inhibits radiation-induced inflammation and fibrotic changes in the salivary gland Expression of phospho-NF-κB, phospho-IκB-α, and TGF-β1 was induced 7, 28, and 56 days after irradiation. phospho-NF-κB and phospho-IκB-α expression levels peaked 7 days after irradiation, whereas TGF-β1 expression peaked 28 days after irradiation. These levels decreased on each day in the ALA-treated irradiated group. β-actin was used as the loading control (**A**). All salivary gland tissue were stained with Masson's trichrome (**B**). Fibrosis signals (blue) were significantly enhanced in irradiated salivary glands, and ALA treatment attenuated the fibrotic changes 28 and 56 days after radiation and lysis of acinar and granular convoluted tubules 7, 14, 28 and 56 days after radiation (**B** and **C**). Con, control. ALA, ALA only. RT, irradiated. ALA + RT, received ALA before irradiation. Scale bar, 50 μm (**C**). Representative blots are derived from three separate experiments. Values are represented as the mean ± SEM. *P < 0.05 and **p < 0.01 indicates differences between RT and ALA + RT groups. The fold changes are calculated as the ratio of the final value in the each group to the value in control group at day 4 (set as “1”). Con (*n* = 3), ALA (*n* = 3), RT (*n* = 10), ALA + RT (*n* = 10).

Radiation-injured SG undergoes a defined pattern of inflammation and fibrosis. The NF-κB signaling pathway is a major source for inflammation.[25] We examined activation of the NF-κB signaling pathway and TGF-β1 expression as a target of the NF-κB signaling. pNF-κB, p-IκB-α, and TGF-β1 protein expression was hardly detectable in the SG 4 days after irradiation in all groups (Figure 5A). Marked induction of pNF-κB, p-IκB-α, and TGF-β1 protein was detected in irradiated SG after 7, 28, and 56 days. pNF-κB and p-IκB-α expression peaked on day 7 in irradiated SG and was maintained until 56 days, whereas, TGF-β1 expression peaked on day 28 in irradiated SG. ALA reduced expression of all of these proteins 4, 7, and 18 days after irradiation. Overexpression of TGF-β1 is associated with fibrosis of the SG [26, 27], which significantly affects saliva secretion [28, 29]. Radiation exposure induces overexpression of TGF-β1 [30]. Consistent with previous observations in irradiated rats [31], only minimal or mild focal fibrosis was observed in the SG of irradiated rats 4 and 7 days after irradiation (Figure 5B). The extent of fibrosis on days 4, and 7 did was no significantly different between rats pretreated with ALA prior to irradiation. However, pretreating rats with ALA clearly resulted in less periductal and perivascular fibrosis than that of untreated SG 28 and 56 days after radiation (Figure 5B and 5C). Lysis of acinar and granular convoluted tubules is also ameliorated by ALA treatment (Figure 5B and 5C).

## DISCUSSION

We conducted biochemical analyses of a variety of signaling pathways at multiple time points and morphological and functional analyses on radiation-induced SG damage. Our data shows time-dependent signaling changes in SG tissue after irradiation. The systemic administration of ALA prior to irradiation improved morphology and functional regeneration, not only in terms of protection against radiation-induced cell death but also decreased oxidative stress, inflammation, and fibrosis. Furthermore, the reduction of apoptosis, inflammation, and fibrosis in ALA-injected SG may be due to a decrease in advanced oxidative stress, as ALA changed gp91 mRNA expression during the acute phase.

One hallmark of radiation-induced SG dysfunction is the lack of recovery in saliva production over time [22]. Most studies show that this dysfunction is chronic [22–25]. In the rat model, radiation-induced suppression of salivary function has been reported to occur almost immediately after exposure, reach a minimum at 3 days, and remain low for 180 days [22, 25]. We also demonstrated that radiation-induced salivary dysfunction reached a maximum 4 days after irradiation. Xu et al. [16] reported that the sharp reduction of saliva flow in mice after irradiation is resulted from overproduction of ROS. Taken together, we suggest that ALA contributes to the intracellular antioxidant system by scavenging ROS, which is involved in the recovery of radiation-induced salivary dysfunction from the early phase.

ROS are involved in SG function impairment by irradiation [20]. Nox is an important ROS source in intracellular signaling pathways [26]. Nox family proteins consist of seven members, such as Nox1, Nox3, Nox4, Nox5, DUOX1, DUOX2, and Nox2/gp91phox [27, 28]. The Nox protein may be considered critical for induction of ROS in irradiated SG due to its expression in SG and ability to generate large amounts of ROS. Nox1 contribute to radiation-mediated ROS production in salivary acinar cells [29]. No studies have shown Nox2 involvement in radiation-induced SG injury. Nox2 (also known as gp91phox) regulates Nox activities by stabilizing p22phox to form its membrane component. Our data show that that ALA decreased gp91 subunit mRNA expression in irradiated SG, suggesting that ALA could prevent radiation-induced salivary oxidative stress at the early stage and thus be a therapeutic option for salivary dysfunction, such as xerostomia.

The extent of apoptosis induced in the SG by head and neck irradiation is causally related to SG dysfunction. [25] In our study, administration of ALA significantly reduced TUNEL-positive signals in salivary ductal cells and acinar cells of irradiated rats 4 and 7 days after irradiation. Apoptotic death at the earlier stages may be due to oxidative stress induced by radiation, as Nox2-derived ROS are involved in ATP-induced apoptosis [28]. The MAPK pathways are important signaling cascades that mediate many cellular functions including apoptosis and inflammation [21, 30]. As shown in Figure 4B, activation of p38 and JNK were increased by radiation and decreased after ALA treatment. Interestingly, these activations were well correlated with expression of gp91 subunit mRNA, 8-OHdG, and apoptotic death. Little is known about the relationship between radiation-induced SG apoptosis and Nox, MAPK, and ROS. Our data suggest that gp91 triggered by radiation might be required for oxidative stress-induced apoptosis through activation of p38 and JNK in irradiated SG.

Activation of p38 and JNK also contributes to induce inflammatory cytokine and chemokines which amplify the inflammatory process at the injury site [31, 32]. Nox-dependent oxidative stress induces the TGF-β1 expression by activating MAPK [33] or through NF-κB.[34–37] Our data show that pNF-κB and p-IκB-α expression decreased significantly by ALA in irradiated rats (Figure 5A). Therefore, we suggest that suppressing NF-κB-mediated salivary inflammation could be considered a critical strategy to ameliorate salivary-dependent side effects in patients with head and neck cancer. Overexpression of TGF-β1 is related with pathological fibrosis of the SG in response to tissue injury.[38–40] We found that TGF-β1 expression in response to radiation decreased significantly by ALA at the subacute phase after irradiation (Figure 5A). Although we cannot explain if TGF-β1 expression is directly related with salivary fibrotic changes by radiation, ALA may have an anti-fibrotic effect in irradiated SG. Finally, our time-dependent data clearly show that ALA acts to prevent radiation-induced oxidative stress that induces salivary cell death and salivary dysfunction through the Nox-2/MAPKs/apoptosis axis at the early phase and reduces responsiveness of radiation-induced fibrosis through the NF-κB/TGF-β1 axis at the subacute period after radiation.

Current management approaches for head and neck cancer are unsatisfactory. ALA is already approved in the clinic. This study is a first step that ALA could prevent the initial loss of salivary function of patients with head and neck cancer. Identifying the pathways mediating Nox2 preservation of salivary function will allow specific targeting of the SGs without modifying tumor therapies. Therefore, current efforts are directed towards building a preclinical model to evaluate the effectiveness of this approach.

## MATERIALS AND METHODS

### Ethics statement

The Gyeongsang National University Institutional Animal Care & ethics committee specifically approved this study (GLA-120120-R0002).

### Radiation exposure

Male Sprague–Dawley rats (230–250 g; Koatech Inc., Peongtaek, Korea) were assigned followings: control (*n* = 12), irradiated (*n* = 16), ALA administered before irradiation (*n* = 16), and only ALA administered (*n* = 12). ALA (100 mg/kg, i.p., Bukwang Pharmaceutical Co., Seoul, Korea) was treated 24 h and 30 min before irradiation. The dose was based on previous studies.[41] The neck area was evenly irradiated with 2 Gy/min (total dose, 18 Gy) using a photon 6-MV linear accelerator (21EX, Varian, Palo Alto, CA, USA). A 3-cm block of Lucite was positioned above the head and neck to provide adequate buildup and facilitate an even radiation distribution. Each rat was exposed to a single dose of radiation and sacrificed 4, 7, 28, and 56 days after radiation.

### Histopathology

Tissues were fixed in 4% paraformaldehyde in 0.1 M PBS, embedded in paraffin, and cut into 5-μm. The sections were stained with hematoxylin and eosin (H & E). Histopathological injury in HE staining has been scored by grading the number of acinar cells with cytoplasmic vacuoles under x 200 magnification field; 0, 0–1; 1, 2–5; 2, 5–10; 3, 10–15; 4, 15–20; 5, > 20. For analyzing the degree of collagen deposition, sections were stained with Masson trichrome (Masson's Trichrome kit, Sigma Diagnostics, St. Louis, MO, USA). SG fields that were randomly selected at x400 magnification were assessed in each rat, and the density of trichrome-positive signals was analyzed using NIS-Elements BR 3.2 (Nikon, Japan).

### Salivary gland function

Salivary functional activity was evaluated by measurement for saliva lag time and secretion. Pilocarpine (1 mg/kg, intraperitoneally, ISOPTO CARPINE, Alcon Korea Ltd, Seoul, Korea)) was injected, lag time was examined as the first time to secret the saliva from pilocarpine stimulation, and 8 minute after stimulation, saliva output was collected from the floor of the mouth for 5 minute. Collected saliva was placed in pre-weighed 1.5 ml microcentrifuge tubes and the volume was normalized to the body weight.

### TUNEL assay

The degree of apoptosis was assessed using TUNEL assay (Indianapolis, IN, USA). Semiquantitative analysis was performed by counting the TUNEL-positive cells per field at 400× magnification. At least 10 areas in the SG per slide were selected randomly. The mean number of brown cells in these selected fields was considered to be the number of TUNEL-positive cells. The signals were analyzed by a blinded observer using NIS Elements BR3.2 (Nikon, Japan) software in 10 randomly selected fields.

### Immunoblot

Tissues were homogenized in lysis buffer. 50 μg of proteins were loaded on a sodium dodecyl sulfate-polyacrylamide gel. The blots were probed with primary antibodies to monoclonal anti-phospho-p38 (Cell Signaling Technology, Danvers, MA, USA), anti-phospho-inhibitor of kappa beta (IκB-α) (Santa Cruz Biotechnology, Santa Cruz, CA, USA), anti-transforming growth factor (TGF)-β1 (Abcam, Cambridge, UK), and polyclonal anti-phospho-JNK (Cell Signaling Technology), and anti-phospho-nuclear factor kappa beta (NF-κB) (Santa Cruz Biotechnology) at 4°C overnight. The primary antibody was visualized using secondary antibodies with an ECL kit (Amersham Pharmacia Biotech, Piscataway, NJ, USA).

### Reverse transcription-polymerase chain reaction (RT-PCR)

Total RNA was extracted from tonsils using the TRIzol method (GIBCO BRL, Grand Island, NY, USA). 5 ug of RNA were converted to cDNA. Reaction products (2.0 μL) were subjected to PCR amplification. The primer sequences are as follows. 5′-TGACTCGGTTGGCTGGCATC-3′ (sense) and 5′-CGCAAAGGTACAGGAACATGGG-3′ (antisense) for gp91. 5′-TCCCTCAAGATTGTCACCAA-3′ (sense) and 5′-AGATCCACAACGGATACATT-3′ (antisense) for GAPDH.

### Immunohistochemistry

After deparaffinization, the sections were incubation with rabbit anti-8-hydroxydeoxyguanosine (8-OHdG) (diluted 1:500; Abcam, Cambridge, MA, USA), with biotin-conjugated secondary IgG (diluted 1:200; Vector Laboratories, Burlingame, CA, USA), with the avidin-biotin-peroxidase complex (ABC Elite kit, Vector Laboratories), and with diaminobenzidine tetrahydrochloride. The sections were visualized under light microscopy, and digital images were captured and analyzed.

### Statistical analysis

Statistical analyses were performed using the Graph Pad Prism 5 (Graph Pad Software Inc., La Jolla, CA. USA). The Mann-Whitney test was used to examine differences between any two groups. A *P*-value < 0.05 was considered significant.

## SUPPLEMENTARY MATERIALS FIGURES


