# Supplementary material for: Protective effects of alpha lipoic acid on radiation-induced salivary gland injury in rats
Source: Oncotarget. 2016 Apr 9;7(20):29143–53. doi: 10.18632/oncotarget.8661 (PMC5045384; doi:10.18632/oncotarget.8661)
Supplement: Supplementary file 1 [file oncotarget-07-29143-s001.pdf]

Protective effects of alpha lipoic acid on radiation-induced salivary gland injury in rats

Supplementary Materials

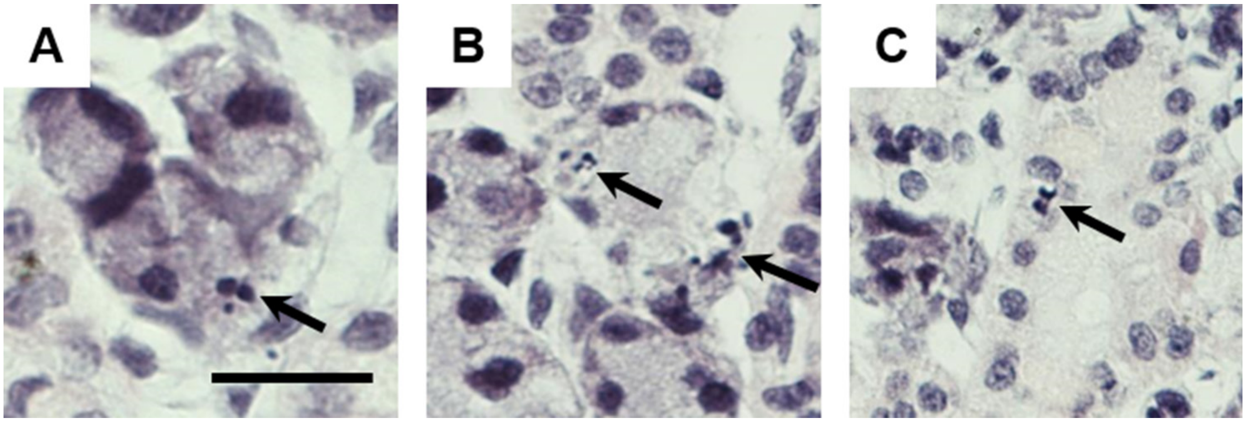

Supplementary Figure S1: Much clear pyknotic nuclei was found in the serous acinar (A), the mucous acinar (B), and the ductal cells (C) in the irradiated SG on 28 day after radiation. Scale bar, 50 µm.

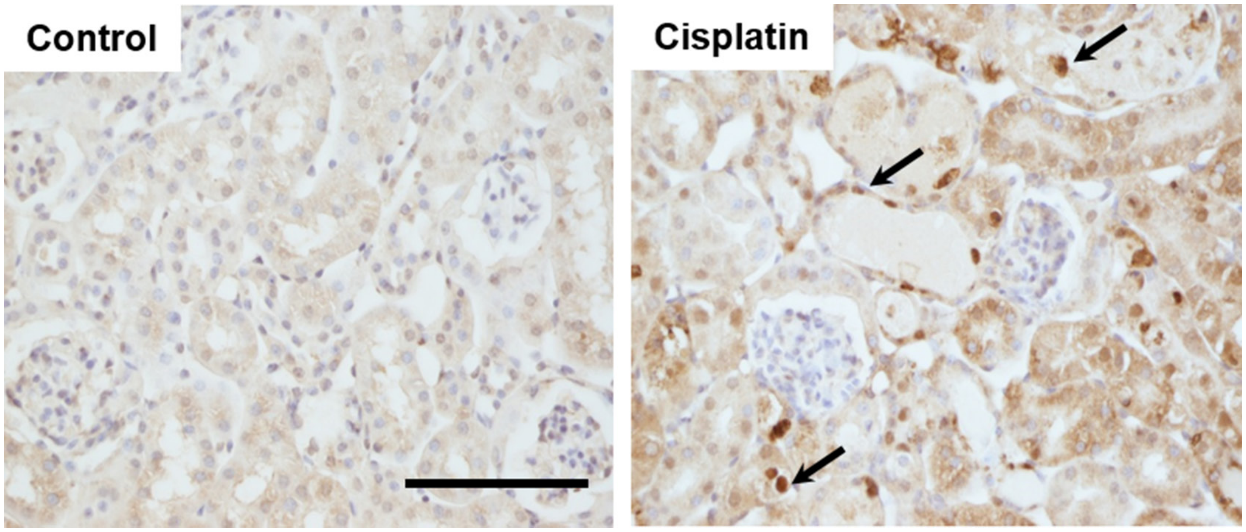

Supplementary Figure S2: To confirm 8-OHdG-positive signals from the irradiated SG, immunohistochemical staining for 8-OHdG was performed in the cisplatin-induced kidney injury in mice model. The expression was clearly found in the renal proximal epithelial cells in the cisplatin-treated kiney. Scale bar, 50 µm.
